# Vulnerability of Left Amygdala to Total Sleep Deprivation and Reversed Circadian Rhythm in Molecular Level: Glut1 as a Metabolic Biomarker

**DOI:** 10.31661/gmj.v8i0.970

**Published:** 2019-06-25

**Authors:** Parastou Kordestani Moghadam, Mohamad Nasehi, Fariba Khodagholi, Mohamad-Reza Zarrindast

**Affiliations:** ^1^Institute for Cognitive Science Studies (ICSS), Tehran, Iran; ^2^Cognitive and Neuroscience Research Center (CNRC), Amir-Almomenin Hospital, Tehran Medical Sciences, Islamic Azad University, Tehran, Iran; ^3^Neuroscience Research Center, Shahid Beheshti University of Medical Sciences, Tehran, Iran; ^4^Department of Pharmacology, School of Medicine, Tehran University of Medical Sciences, Tehran, Iran

**Keywords:** Sleep, Circadian Rhythm, Glucose Transport Protein, Amygdala

## Abstract

**Background:**

Sleep deprivation (SD) in the long term can cause multi-organ dysfunction as well as neurocognitive disorders. Daytime sleep or napping is a biological compensate due to insomnia or sleep deprivation. Metabolic responses to this biological rhythm may being as a biological indicator or biomarker to compare the effect of them. Glucose transporter type 1 (Glut1) is one of the metabolic biomarkers that is affected by several conditions such as stress, seizure, malignancy, and neurocognitive disorders. We studied the effect of SD, circadian reversed (R) and napping models on the Glut-1 expression level in the right and left amygdala.

**Materials and Methods:**

Sixty-four Wistar rats were divided into eight groups as follow: Intact group that rats were placed in a cage without any intervention. In the sham group, rats were on the stable pedal of the SD apparatus (turn off). Experimental groups include total SD48, total SD48- (plus short nap), total SD48+ (plus long nap), R48, R48- (plus short nap), and R48+ (plus long nap). The Glut-1 expression level in the right and left amygdala were measured by western blotting.

**Results:**

Our findings demonstrated the significant effect of both SD for 48 hours and reversed circadian on the expression of Glut-1 from sham and intact groups. The long nap plus them could decrease the elevation of Glut-1 in the left amygdala. However, the short nap could not reduce this elevation of Glut-1.

**Conclusion:**

Left amygdala is vulnerable to the fluctuation of hypothalamic-pituitary-adrenal axis and stress. In other words, sleep disorders are affecting by Glut-1 as a metabolic biomarker in left amygdala alone.

## Introduction

Regarding technology advancement and urbanization, sleep deprivation (SD) and sleep disorders are considered as one of the most important health problems in all around the world [1]. SD in the long term can cause multi-organ dysfunction like hypertension, obesity, diabetes, depression, heart attack, and cerebrovascular accident as well as neurocognitive disorders [2]. In general, sleep has a special role in mental health and adaptation throughout life [3]. Many evidences emphasize the role of sleep in the emotional memory consolidation [4, 5]. Evaluation and emotional appraisal are happening as silence in sleep [1]. Memory processing and consolidation are one of the physiological events that occur off-line in sleep especially in rapid eye movement (REM) sleep. Working memory and daily events enhance and establish at this time. In the case of every one experiences non-REM (NREM) sleep perfectly, memory consolidation and improvement happens. On the other hand, REM sleep consolidates emotional memory [6, 7]. One of the compensatory mechanism due to SD is napping. There is controversial evidence of napping including of negative effects of a long nap because the subject experiences slow wave sleep, then wakefulness after this sleep stage is trouble so can decrease of brain performance [8-10]. Meanwhile, the short nap is including of the REM sleep stage, then can consolidate emotional memory [11]. Thus, it seems that the short nap has the better effect than a long nap, but it wasn’t established. Despite the findings of previous studies, there are several questions about the positive and negative effects of the kind of nap. The most abundant neurotransmitter in brain synapses is glutamate (GLU). It is interesting that 80 percent of brain synaptic neurotransmitters assign to GLU as inside or outside of synapses. Release and potentiation of GLU from synaptic vesicle is an essential role of astrocytes as well as catabolism of extracellular GLU by neuroglia. GLU in astrocyte is catabolized to glutamine (glutamine synthetase), alanine (alanine aminotransferase), alpha-ketoglutarate (GLU dehydrogenase) and glutathione [12-14]. There is the important issue that GLU metabolizing to glutamine is essential for the maintaining normal activity of GLUergic synapses [15]. One outcome of GLU catabolism is the promotion of antioxidant synthesis. The activity of antioxidants plays the main role in preventing of neurodegenerative disorder like as Alzheimer and Parkinson diseases [16].Many studies were demonstrated that the level of enzymes and proteins involved in the Krebs cycle are altered due to insomnia. It’s clear that metabolic disorders appear due to Krebs cycle dysfunction [17, 18]. The process of neural metabolic in the brain depends on astrocytes activity that produces lactate as well as activates glycolysis and glycogen metabolism. The astrocyte end-feet that may cover entirely the capillary surface can show the presence of glucose transporter type 1 (Glut-1) the site of glucose uptake. Furthermore, astrocytes can connect to capillaries, and on the other site are linked with neurons and synaptic processes. The concept of brain metabolism is based on this integration of astrocytes and neurons [19]. Based on the previous studies, increasing of Glut-1 is a biomarker in multiple brain disorders such as malignancies [20], stressful situation, depressed mood [21]. Deficiency of Glut-1 caused in the other brain dysfunctions like as paroxysmal dyskinesia due to exercise, absence seizure and Glut-1 deficiency syndrome [22]. Increasing levels of stress and chronic stress as an allostatic load are overthrown and body systems dissociation that they induced in brain structure alteration excess clinical manifestations. In animal model studies, chronic stress causes the spectrum of the alteration in the prefrontal cortex as well as hippocampus neuronal atrophy, which is involved in memory and executive function. But hypertrophy is seen in the amygdala that is involved in stress and emotions [1]. Actually, SD and stress aren’t two separated issues. The range of the level of stress is included in sleep, drowsy to wakefulness, alertness and motivated wakefulness optimized in physical stress and emotional arousal. As SD subjects with physical or brain activity by applying attention had a higher activity autonomous system and experienced the high level of stress [23]. With regard to evidence about the effect of stress and SD as the well as the effect of short and long nap on the metabolic and behavioral function of the brain, specially amygdala, this study was accomplish to determine the effect of SD, reversed circadian and napping models on the expression of Glut-1 in the right and left amygdala.

## Materials and Methods

### 1. Animals

In this study, 64 male Wistar rats, weighing 200 to 250 g were used. The animals were maintained in standard laboratory conditions at 22±3°C. Light-dark cycle of 12 hours light and 12 hours of darkness were established. Animals were 48 hours under conditions of SD, napping or reverse insomnia. The conditions for animals on the same day or the light cycle were carried out during the experiments, the animal’s food and water were sufficient. Behavioral testing was performed in the process of lighting.

### 2. SD Apparatus

**Figure 1:**
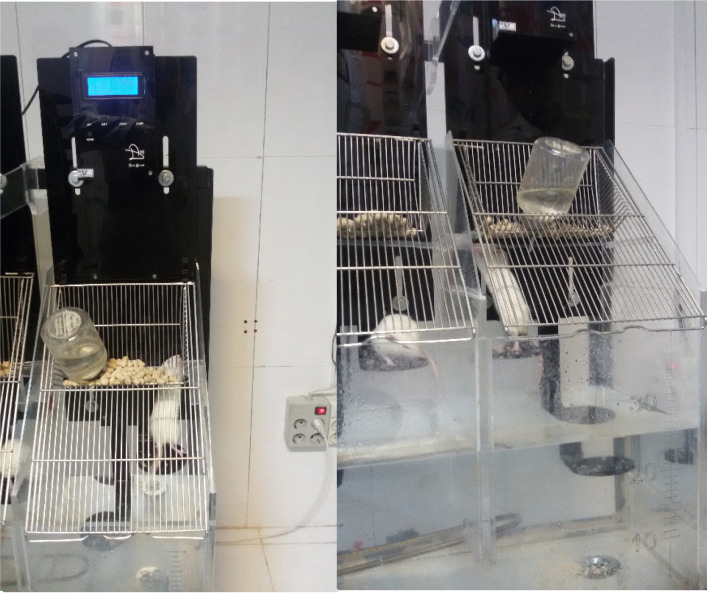
SD apparatus ‘‘water-box”. A holding time that was considered to allow water and food intake.

The SD digital device (Figure-1) with adjustable parameters used so that the animal was placed on a pedal based Weber timer on the device was set, the pedals move, and animals should exercise caution before entering into the water under the pedals, their subsequent transfer to the pedals [24]. This process causes insomnia. It should be noted that the setting napping model on the same machine was possible.

### 3. SD Models

#### 3.1. Total SD Model

Animals were placed in sleep deprivation apparatus for 48 hours on pedals while are moving.

#### 3.2. Reverse Circadian Rhythm Model

In reverse circadian rhythm model or R48 model, animals were placed for 48 hours. Rats were deprived of sleep12 hours in any 24-hour reverse or opposite circadian rhythm. In this model, animals were deprived of sleep from 7 am to 7 pm insomnia.

### 4. Napping Models

#### 4.1. Short Nap

Animals were allowed to sleep every 20 minutes for 3 minutes. This opportunity during the total and reverse insomnia, each one was separately applied.

#### 4.2. Long Nap

Animals were allowed to sleep for 20 minutes every 3 hours. This opportunity during the total sleep deprivation and reverse circadian insomnia, each one was separately applied.

### 5. Grouping

All the rats were randomly divided into eight groups (n=8 per group) as follow:

Intact group that rats were placed in a cage without any intervention. In the sham group, rats were on the stable pedal of the SD apparatus (turn off). Experimental groups include total SD48, total SD48- (plus short nap), total SD48+ (plus long nap), R48, R48- (plus short nap), and R48+ (plus long nap).

### 6. Extracted Brain Tissue and the Nucleus of the Left and Right Amygdala

After finishing SD and napping models, the animals were placed in a special chamber and then be killed with CO2 gas. The brain was removed. After a brief rinse with normal saline in proper brain matrix rats weighing 250-200g, cut by a razor-thin coronal created and nucleus of the hypothalamus in the bottom right and left separately isolated and encoded separately in microtubes were placed and then inserted the nitrogen tank. All tissue extraction procedure should not last more than 2 minutes. After 24 hours the samples were transferred from the tank to the freezer -80°^c^.

### 7. Western Blotting

Western blotting was used to detect the Glut-1 level. For this aim at first, left and right amygdala tissues separately were homogenized in special lysis buffer (containing Tris-HCl, NaCl, sodium deoxycholate, sodium dodecyl sulfate, EDTA, Triton X 100, and protease inhibitor cocktail). The protein level of each sample determined by the Bradford method [25]. Same volume (60 micrograms) of protein was loaded on a 12% polyacrylamide gel and then transferred to a polyvinylidene difluoride membrane. The membranes were blocked using nonfat milk (blocking buffer) and then incubated with primary antibody against Glut-1( Cell signaling company; USA). After that, blots were incubated with an HPR-conjugated secondary antibody ( Sigma Company; Germany) Immunoreactive proteins were detected using enhanced chemiluminescence reagents (Sigma Company; Germany). β-actin antibodies ( Sigma Company; Germany)were used to confirmation of equal loading of protein. Densitometry carried out by Image J software (ImageJ bundled with 64-bit Java 1.8.0_112; NIH; USA), and relative density of protein bands was determined.

### 8. Ethical Statement

The experimental protocol was done in accordance with the National Institutes of Health Guide for the Care and Use of Laboratory Animals (NIH publications No. 80–23).

### 9. Statistical Analysis

One-way analysis of variance (ANOVA) and Tukey’s post hoc tests were used as a statistical test for comparing Glut-1 between groups and independent t-test to compare Glut-1 between the two brain hemispheres (right and left Amygdala) by the 5^th^ edition of GraphPad Prism software ( 6^th^ version) .

## Results

### Comparing the level of Glut-1 Between Groups in the Left Amygdala

**Figure 2:**
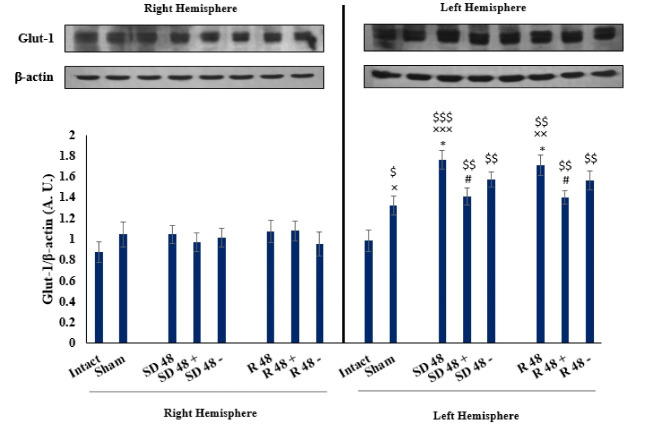
Comparison of Glut-1 level between groups and among two hemispheres (right and left amygdala). *P<0.001 vs. Sham group; ×P<0.05, ××P<0.01, and ×××P<0.001 vs. intact group; #P<0.01 vs. SD48; $P<0.05, $$P<0.01, $$$P< 0.001and differences between two hemispheres.

The Glut-1 levelin the SD48 group showed a significant statistical difference compared to sham and intact groups (P<0.001, Figure-2). Also, R48 demonstrated a significant difference compared to intact and sham groups (P<0.001 and P<0.01, respectively). SD48+ showed a significant difference compared to the SD48 as well as the R48+ to R48 group (P<0.01). Furthermore, the other groups have not a meaningful difference between them.

### Comparing the level of Glut-1 Between Groups in the Right Amygdala

There was no any significant statistical difference between the groups on the right side (Figure-2).

### Comparing Two Hemispheres in Each Group

There was a significant difference between the left and right amygdala in the SD48 group (P<0.001) and was established in the other experimental groups such as SD48+, SD48-, R48, R48+, R48- groups (P<0.01) and beside in the sham group (P<0.05). Meanwhile, there was no any significant difference between the two sides in the intact group.

## Discussion

The findings of this study were demonstrated that in the left amygdala, total SD and reversed circadian for 48 hours could increase the expression of the Glut-1 beside sham and intact groups. Some studies showed the SD is a stress in the brain and the other organs. In other words, any alteration in biological rhythm is equal the stress for all body systems [23, 26]. The brain is the first impressionable organ from stress and SD. In the meantime, the amygdala has a key role in emotional processing and stress reduction; it seems that amygdala involvement is more than the other area of the brain [27]. Hereupon, “first night effect” is a phenomenon about a new place to sleep that is defined by the trouble to sleep and a higher level of electrophysiological arousal or decrease of depth of sleep (reduction of slow-wave sleep) in the one brain hemisphere than the other hemisphere [28]. In this study, the rise of the Glut-1 level in the left amygdala in experimental groups represents the increase of metabolism in the left amygdala and in the other word decrease of sleep depth or higher arousal in the amygdala, which has a central role in emotion. Thus, it seems that the kind of arousal under the label “emotional arousal.“ Regarding this evidence, the left amygdala is more vulnerable to stress and SD than the right amygdala. Interestingly, long nap model plus SD48 could decrease the Glut-1 expression in the left amygdala beside the SD48 alone, as well as theR48 plus long nap compares to the R48 alone. The previous studies demonstrated that a decrease in stress could modulate the hypothalamic pituitary adrenal (HPA) axis and hormonal pathway that increase of metabolic activity [23]. Attending to the asymmetry of brain activity within molecular, functional, and behavioral to response the stress and SD [29] because the left amygdala showed higher activity in the metabolic system, It seems that every area is more sensitive to insomnia should have a better response to cure in the acute phase. Instead, short nap plus each SD and reversed circadian, have not a significant effect on the expression of Glut-1 compare to anyone alone. It seems that repeated wakefulness can increase the level of stress hormone (cortisol) and activate HPA axis [23] and ultimately the stress-induced rise of glucose and glycolysis. Drevets *et al*. in the study of glucose metabolism in the amygdala in depressed patients has demonstrated the significant positive correlation between the left amygdala metabolism and the plasma cortisol level [26]. Furthermore, Ledoux in the study of depressed patients found a significant positive correlation between depression severity and amygdala blood flow [27]. Follow the long time stress and the rise of demand to glucose to produce ATP for GLUergic neuronal activity especially in the amygdala; it’s anticipated to consume even overuse of glucose causes a decrease of glucose against the rise of glucose transporters like Glut-1 [21]. Based on compensatory mechanisms that happened in a short time, it may cause to reduce the level of Glut-1 due to chronic stress as well as SD and reversed circadian and any situation, which induced irregular rhythm in body organ system. Finally, every person experienced the same condition, may progress to exhaustion and some disorders follow them such as Alzheimer, Parkinson disease, and the other neurodegenerative disease. But, in the right amygdala, the findings in experimental groups have no any significant difference from the sham and intact groups. Attending to previous studies, right brain hemisphere especially amygdala is lower vulnerable to stress and insomnia than the left side.

## Conclusion

Attending to the vulnerability of the left amygdala to SD in this study, we have some suggestions for people that experience SD or insomnia. One of them is decreasing the stimulator that activates the left amygdala, like loud sounds, physical activity, and the other stresses. Use of the long nap during the night for the employees that work at night and experience some stressors, for example, the nurses should long nap for protection their brain and enhance the cognitive performance to prevent of medical errors. Also, we suggest the long nap should consist of one cycle of REM and NREM. Finally, it prefers the people that suffer from seizure, dyskinesia, depression, anxiety-like behavior, dementia, and metabolic disorders avoid the SD situations. Indeed, they are more vulnerable to SD than the other persons due to Glut-1 alteration.

## Acknowledgment

The authors of this paper appreciate and acknowledge the sleep lab of the Institute for Cognitive Science Studies and Neuroscience, Neurobiology lab of Shahid Beheshti University of medical science for close collaborations.

## Conflict of Interest

The authors declare there is no any conflict of interests.

## References

[1] McEwen BS (2006). Sleep deprivation as a neurobiologic and physiologic stressor: Allostasis and allostatic load. Metabolism.

[2] Benedetti F, Colombo C (2011). Sleep deprivation in mood disorders. Neuropsychobiology.

[3] Palmer CA, Alfano CA (2017). Sleep Architecture Relates to Daytime Affect and Somatic Complaints in Clinically Anxious but Not Healthy Children. J Clin Child Adolesc Psychol.

[4] Payne JD, Kensinger EA (2011). Sleep leads to changes in the emotional memory trace: evidence from FMRI. J Cogn Neurosci.

[5] Payne JD, Stickgold R, Swanberg K, Kensinger EA (2008). Sleep preferentially enhances memory for emotional components of scenes. Psychol Sci.

[6] Tucker MA, Hirota Y, Wamsley EJ, Lau H, Chaklader A, Fishbein W (2006). A daytime nap containing solely non-REM sleep enhances declarative but not procedural memory. Neurobiol Learn Mem.

[7] Wulff K, Gatti S, Wettstein JG, Foster RG (2010). Sleep and circadian rhythm disruption in psychiatric and neurodegenerative disease. Nat Rev Neurosci.

[8] Akerstedt T, Torsvall L (1985). Napping in shift work. Sleep.

[9] Watamura SE, Donzella B, Kertes DA, Gunnar MR (2004). Developmental changes in baseline cortisol activity in early childhood: relations with napping and effortful control. Dev Psychobiol.

[10] Takahashi M (2003). The role of prescribed napping in sleep medicine. Sleep Med Rev.

[11] Walker MP, van der Helm E (2009). Overnight therapy? The role of sleep in emotional brain processing. Psychol Bull.

[12] Marcaggi P, Attwell D (2004). Role of glial amino acid transporters in synaptic transmission and brain energetics. Glia.

[13] Sofroniew MV (2009). Molecular dissection of reactive astrogliosis and glial scar formation. Trends Neurosci.

[14] Morales I, Rodriguez M (2012). Self-induced accumulation of glutamate in striatal astrocytes and basal ganglia excitotoxicity. Glia.

[15] Featherstone DE (2010). Intercellular glutamate signaling in the nervous system and beyond. ACS Chem Neurosci.

[16] Darby M, Kuzmiski JB, Panenka W, Feighan D, MacVicar BA (2003). ATP released from astrocytes during swelling activates chloride channels. J Neurophysiol.

[17] Starkov AA, Fiskum G, Chinopoulos C, Lorenzo BJ, Browne SE (2004). Mitochondrial alpha-ketoglutarate dehydrogenase complex generates reactive oxygen species. J Neurosci.

[18] Tretter L, Adam-Vizi V (2005). Alpha-ketoglutarate dehydrogenase: a target and generator of oxidative stress. Philos Trans R Soc Lond B Biol Sci.

[19] Falkowska A, Gutowska I, Goschorska M, Nowacki P, Chlubek D, Baranowska-Bosiacka I (2015). Energy Metabolism of the Brain, Including the Cooperation between Astrocytes and Neurons, Especially in the Context of Glycogen Metabolism. Int J Mol Sci.

[20] Young CD, Lewis AS, Rudolph MC, Ruehle MD, Jackman MR (2011). Modulation of glucose transporter 1 (GLUT1) expression levels alters mouse mammary tumor cell growth in vitro and in vivo. PLoS One.

[21] Detka J, Kurek A, Basta-Kaim A, Kubera M, Lason W, Budziszewska B (2014). Elevated brain glucose and glycogen concentrations in an animal model of depression. Neuroendocrinology.

[22] Weber YG, Kamm C, Suls A, Kempfle J, Kotschet K (2011). Paroxysmal choreoathetosis/spasticity (DYT9) is caused by a GLUT1 defect. Neurology.

[23] Meerlo P, Sgoifo A, Suchecki D (2008). Restricted and disrupted sleep: effects on autonomic function, neuroendocrine stress systems and stress responsivity. Sleep Med Rev.

[24] Norozpour Y, Nasehi M, Sabouri-Khanghah V, Torabi-Nami M, Zarrindast MR (2016). The effect of CA1 alpha2 adrenergic receptors on memory retention deficit induced by total sleep deprivation and the reversal of circadian rhythm in a rat model. Neurobiol Learn Mem.

[25] Bradford MM (1976). A rapid and sensitive method for the quantitation of microgram quantities of protein utilizing the principle of protein-dye binding. Anal Biochem.

[26] Drevets WC, Price JL, Furey ML (2008). Brain structural and functional abnormalities in mood disorders: implications for neurocircuitry models of depression. Brain Struct Funct.

[27] LeDoux J (2003). The emotional brain, fear, and the amygdala. Cell Mol Neurobiol.

[28] Tamaki M, Bang JW, Watanabe T, Sasaki Y (2016). Night Watch in One Brain Hemisphere during Sleep Associated with the First-Night Effect in Humans. Curr Biol.

[29] Ocklenburg S, Korte SM, Peterburs J, Wolf OT, Gunturkun O (2016). Stress and laterality - The comparative perspective. Physiol Behav.

